# Inheritance of Early Stomatal Closure Trait in Soybean: Ellis × N09-13890 Population

**DOI:** 10.3390/plants12183227

**Published:** 2023-09-11

**Authors:** Avat Shekoofa, Victoria Moser, Kripa Dhakal, Isha Poudel, Vince Pantalone

**Affiliations:** 1 Department of Plant Sciences, University of Tennessee, 2505 E.J. Chapman Dr., Knoxville, TN 37996, USAvpantalo@utk.edu (V.P.); 2 FedEx Institute of Technology, University of Memphis, 365 Innovation Drive, Suite 228, Memphis, TN 38152, USA

**Keywords:** fraction of transpirable soil water (FTSW), recombinant inbred lines (RILs) soybean, water-deficit stress

## Abstract

Drought conditions exhibit various physiological and morphological changes in crops and thus reduce crop growth and yield. In order to mitigate the negative impacts of drought stress on soybean (*Glycine max* L. Merr.) production, identification and selection of genotypes that are best adapted to limited water availability in a specific environmental condition can be an effective strategy. This study aimed to assess the inheritance of early stomatal closure traits in soybeans using a population of recombinant inbred lines (RILs) derived from a cross between N09-13890 and Ellis. Thirty soybean lines were subjected to progressive water-deficit stress using a dry-down experiment. The experiment was conducted from June to November 2022 at the West Tennessee Research and Education Center (WTREC), University of Tennessee in Jackson, TN, under controlled environment conditions. This study identified significant differences among soybean lines in their early stomatal closure thresholds. The fraction of transpirable soil water (FTSW) thresholds among 30 tested lines ranged from 0.18 to 0.80, at which the decline in transpiration with soil drying was observed. Almost 65% of the RILs had FTSW threshold values between 0.41 to 0.80. These results, indicating inheritance, are supportive of the expression of early stomatal closure trait in progeny lines at a high level in cultivar development for water-deficit stress conditions. Thus, identifying the differences in genotypes of water use and their response to water-deficit stress conditions can provide a foundation for selecting new cultivars that are best adapted to arid and semi-arid agricultural production systems.

## 1. Introduction

Crop growth and yield are limited by extreme weather events such as rising temperatures, unpredictable rainfall patterns, and droughts, which are major environmental stresses [1,2]. Among these, water-deficit stress is one of the most important physiological factors that adversely affect the growth, productivity, and metabolism of soybeans (*Glycine max* L. Merr.) [3]. Soybean is a major legume crop with immense economic significance [4,5,6], but its production is highly dependent on optimum rainfall or irrigation [6]. In the Southeastern U.S., drought is the leading cause responsible for soybean yield reduction [4,5,6]. Hence, strategies that make plants more resilient to stressors are needed to ensure sustained productivity under current and future climates.

One potential trait that could confer drought tolerance in soybeans is the response of transpiration rate (TR) to progressive soil drying [7,8,9,10,11]. Water-deficit-stressed plants undergo anatomical, physiological, and biochemical changes that result in reduced water uptake and increased water loss via transpiration. Nonetheless, they respond by activating physiological mechanisms such as early stomatal closure and decreased transpiration rate [12,13].

Stomatal conductance plays a vital role in regulating plant CO_2_ uptake and water loss, significantly influencing the cumulative rate of photosynthesis and water use throughout the growing season [14]. Hence, early decreases in stomatal conductance under drought conditions is a promising physiological trait for developing drought-resistant soybean genotypes. Previous studies have shown that plants with low transpiration rate partially close their stomata under water-deficit stress conditions, conserving more water in the soil for the later stages of growth [15]. Several physiological traits such as transpiration rate, leaf expansion, leaf epidermal conductance, leaf area, and photosynthesis have been examined in previous studies to evaluate drought tolerance in soybean [11,16].

When the soil dries up, plants respond by decreasing their transpiration rate, and this point is referred to as the fraction of transpirable soil water (FTSW) threshold. The higher FTSW threshold values exhibit significant sensitivity to early soil drying, which implies a potential for soil water conservation and improved drought tolerance [17]. Sadras and Milroy reported that most crop plants, including soybeans, have FTSW thresholds for a decrease in transpiration rate within the range of 0.25 to 0.40 [18]. However, studies have shown significant variation in the FTSW threshold among different crops and species. 

For example, Devi et al. compared 17 peanut genotypes for their transpiration response to drying soil and found a wide range of FTSW threshold values, ranging from 0.22 to 0.71 [8]. Another study comparing 24 soybean genotypes showed a substantial variation in FTSW threshold values grown in two different potting media, sandy soil (80% sand) and earth potting mix; the FTSW threshold ranged from 0.22 to 0.29 and 0.19 to 0.27, respectively [19]. Furthermore, the FTSW threshold value was found to be affected by the ancestral soybean (*Glycine soja*) growing at two different temperatures, with threshold values of 0.28 and 0.67 observed at 30 and 25 °C, respectively [20].

In order to screen for drought tolerance traits, it is crucial to carefully select appropriate parents for crossing. One such cultivar is Ellis [21], a drought-tolerant cultivar of soybean [11,22] developed by the University of Tennessee, Agricultural Experiment Station, TN. A previous study found that Ellis expressed an FTSW threshold within the range of 0.59 to 0.69, indicating that the plant starts closing its stomata, reducing the transpiration rate in response to progressive soil drying [11]. In addition, Ellis showed delayed wilting under water deficit conditions in the field [22]. Another line, N09-13890, is a slow wilting line with a high water use efficiency, biomass, and seed yield but has reduced root development with more resources allocated for shoot growth [23]. 

The recent availability of the entire soybean genome sequence and genome-wide expression profiling data makes it possible to identify key genes regulating drought tolerance. However, the identification and use of traits related to drought tolerance and the development of suitable screening techniques are the prime criteria for cultivar development [24].

The objective of this study was to assess the performance of 30 recombinant inbred lines (RILs) derived from the cross between Ellis and N09-13890 soybean lines under water-deficit stress conditions. The study aimed to investigate the inheritance of early stomatal closure, a drought tolerance trait, and identify the most promising RILs based on their transpiration response to progressive soil drying.

## 2. Results

### 2.1. Expression of the TR Trait

**Figure 1:**
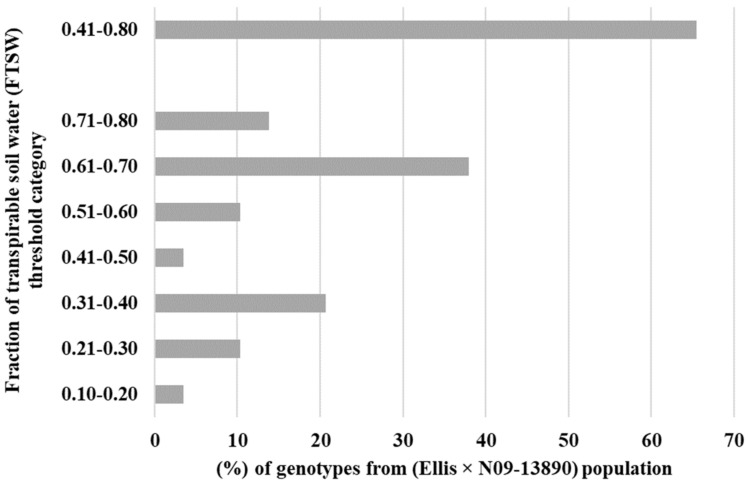
Percentage of tested lines in each fraction of transpirable soil water (FTSW) category. A little over 65% of tested lines from crossing Ellis and N09-13890 placed in the 0.41–0.80 category.

**Figure 2:**
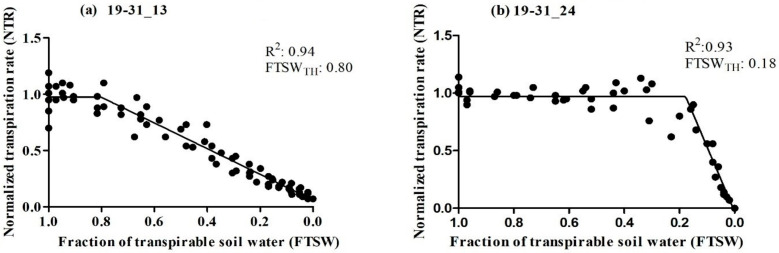
Relationship between normalized transpiration rate (NTR) plotted against the fraction of transpirable soil water (FTSW) of two soybean lines tested (**a**) 19-31_13 and (**b**) 19-31_24. The breakpoint in the graph indicates the amount of water remaining in the soil when the plants start to partially close their stomata.

The primary objective of this study was to assess the transpiration rate response of 30 RILs. We hypothesized that some of the RILs may have drought tolerance traits inherited from their parental lines (Ellis and N09-13890), specifically with regard to the early stomatal closure trait during soil dry-down. The results indicated significant variability in the FTSW threshold among the RILs, ranging from 0.18 to 0.80 (Table 1). Ellis exhibited an FTSW threshold between 0.61 and 0.70, which was surpassed by several other RILs. Multiple lines had a higher value of FTSW than their parental line, Ellis. Approximately 13% of the RILs had FTSW threshold values between 0.71 and 0.80, indicating that the combination of the two parental lines had a positive impact on the expression of drought tolerance traits in the progeny (Figure 1). Almost 65% of the Ellis × N09-13890 tested population had FTSW threshold values between 0.41 and 0.80 (Figure 1). All lines showed changes in NTR as the soil dried, as demonstrated using the two-segment linear regressions with an R^2^ coefficient ranging from 0.83 to 0.99 (Table 1). This indicated that progressive soil drying was a reliable indicator of the transpiration rate response among RILs. The FTSW threshold values for NTR were compared among soybean lines based on their 95% confidence intervals (Table 1). Two lines, 19-31_13 and 19-31_24, exhibited the highest and lowest FTSW threshold values, with 0.80 and 0.18, respectively (Figure 2). It was observed that lines with higher FTSW threshold values had the earliest stomatal closure and reduced transpiration rate.

### 2.2. Transpiration Rate Response to Vapor Pressure Deficit (VPD)

**Figure 3:**
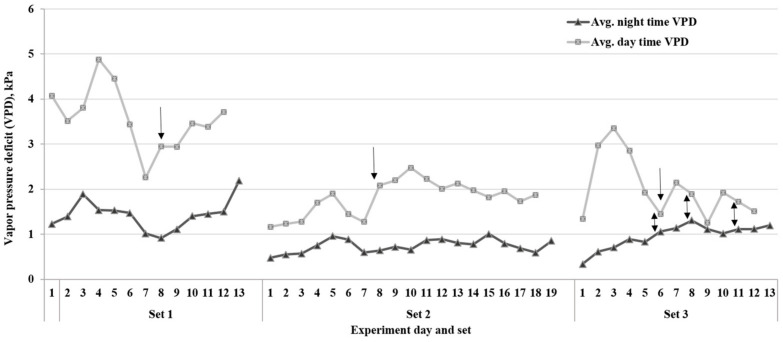
Average daytime and nighttime vapor pressure deficit (VPD) on each day during the three sets of dry-down experiments. Arrows show the day that plants started reaching the fraction of transpirable soil water (FTSW) thresholds for the transpiration rate. The two-way arrows show the small gap between day and night VPD rates in set 3 of the study.

The duration of the experiment varied among the three sets, and lines took different lengths of time to reach the endpoint of the drying down cycle (Table 2 and Figure 3). These differences were likely due to the environmental variables such as temperature and VPD during each set of experiments. The average VPD during the period when FTSW threshold values were reached in set 1 was ~3.0 kPa. The other two sets, 2 and 3, had 2.0 and 1.3 kPa VPD rates, respectively. Based on Tukey’s HSD test (*p* < 0.05) of the average for each of the three experiments, there was a significant difference in the VPD between set 1 and the other two sets (Figure 3). As a result, the drying down period was longer in set 2 for most lines due to the low evaporative demand rates compared to set 1 of the experiment. Additionally, the FTSW threshold values for all lines in set 3 were lower than the other two sets (Table 1).

## 3. Discussion

Plants have developed complex mechanisms to cope with water-deficit stress, including modifications in gene expression, osmotic adjustment, stomatal regulation, and root architecture [25]. These mechanisms are regulated using a complex network of genes, and their expression is influenced by various environmental factors, including temperature, humidity, soil type, and nutrient availability [26,27]. 

In recent years, the fraction of transpirable soil water (FTSW) threshold responses to transpiration rate have been widely used to assess plant water loss responses under water deficit conditions [10,11,28]. The objective of this study was to assess the performance of 30 recombinant inbred lines (RILs) derived from the cross between Ellis and N09-13890 soybean lines under water-deficit conditions. We investigated the line’s FTSW threshold values for the decrease in NTR during progressive soil water drying experiments. There were considerable differences in the range of thresholds among 30 RILs across experiments (Table 1). 

Based on their FTSW values, more than half of the lines had an FTSW threshold equal to or greater than that of the parental line, Ellis [11,22], which was used in the population’s development. These findings imply that the combination of genes inherited from both parental lines had a positive effect on the progeny, whereas the N09-13890 parent contributed to the expression of limited transpiration rate (TR_lim_), another drought tolerance trait [23]. Therefore, a deep understanding of the physiological basis for stomatal regulation is required. An important parameter, transpiration efficiency (TE), is under genetic control and can reduce the amount of water loss under water-limited environments [24].

When plants experience long-term water-deficit stress, it adversely reduces photosynthetic metabolism by limiting stomatal operations [29]. Stomatal closure decreases the concentration of CO_2_ required in the carboxylation process in the mesophyll during photosynthesis. While this can reduce the rates of photosynthetic metabolism, it also helps to conserve water and minimize the transpiration rate, which is essential for not only survival but also productivity of crops during prolonged periods of drought. The number, size, opening, and arrangement of stomata on the leaf surface all influence stomatal conductance [16]. One previous study reported that water-stressed soybean cultivars exhibited a low density of stomata [3], and there were significant variations in stomatal density among the various genotypes.

The current study revealed that there are genotypic variations in the transpiration rate response of soybean RILs under water-deficit stress. The results indicated that some lines react faster than others to save water during the rise of average VPD during the period when FTSW threshold values were reached. The experiment with higher VPD rates (sets 1 and 2) (Figure 3) had a higher range of FTSW threshold values (Table 1) compared to set 3. The high VPD results in stomatal closure in certain soybean lines with the limited transpiration trait, which leads to soil water conservation [9,30]. 

The high percentage of RILs (i.e., 65%) (Figure 1) placed in 0.41 to 0.80 FTSW threshold values category in the progeny lines suggests that it is possible to develop drought-resistant soybean lines from crossing drought tolerant lines with expression of varying drought tolerance traits [11,22,23]. Thus, these results are encouraging in that crossing in a breeding program with a parent (i.e., Ellis) that expressed both early stomatal closure under soil drying as well as air drying, high VPD.

These findings demonstrated that genotypic variation existed in soybean RILs. The results emphasize the importance of considering genotypic variation in the selection of drought-resistant soybean lines concerning transpiration rate response, especially for production under diverse environmental conditions. By targeting stomatal traits that differ between lines, breeders could develop more water-efficient lines that can maintain or increase yield under a range of environmental conditions.

## 4. Materials and Methods

### 4.1. Plant Material and Growth Condition

A population of recombinant inbred lines (RILs) was developed from a cross in 2019 between N09-13890 as the female and Ellis as the male at the East Tennessee Research and Education Center (ETREC) in Knoxville, TN. The F1 seeds were grown under lights at the United States Department of Agriculture (USDA) Tropical Agricultural Experiment Station in Isabela, Puerto Rico, and the F2 seeds were planted at ETREC in 2020. The F3 seeds were harvested by picking one pod per plant and grown in an unlighted nursery at 3rd Millennial Genetics in Santa Isabel, Puerto Rico, during the winter of 2020–2021. 

The subsequent seed was harvested after maturity using single pod descent, and the F4 seeds were planted at ETREC in the summer of 2021; approximately 300 single F4 plants were selected to grow the F4:5 in 2022. Among them, thirty lines were randomly chosen and tested for their transpiration rate response to progressive soil drying in a controlled environment at the West Tennessee Research and Education Center (WTREC), University of Tennessee in Jackson, TN. 

The soybean lines were grown in a greenhouse in 3.8-L pots filled with a soil mixture of fifty percent sand and fifty percent Lexington silt loam (fine-silty, mixed, active, thermic Ultic Hapludalf). After the emergence of the unifoliate leaf, plants were thinned to one per pot. Thirteen and twenty days after planting, pots were fertilized with 200 mL of 0.075% *v*/*v* liquid fertilizer (0-10-10, N-P_2_O_5_-K_2_O, GH Inc., Sebastopol, CA, USA). Temperature and relative humidity in the greenhouse were recorded every five minutes with USB data loggers (Lascar Electronics Ltd., Erie, PA, USA). The recorded air temperature and relative humidity data were used to calculate the vapor pressure deficit (VPD, kPa). Vapor pressure deficit can be defined as the amount of vapor that can still be stored in the air until reaching saturation point under the same temperature. This variable can be calculated as the difference between the actual vapor pressure and the saturation vapor pressure [10]. 

First, the saturation vapor pressure (SVP, kPa) was calculated:

SVP = 0.6108 × exp (T/(T + 237.3) × 17.2694)

T: degrees Celsius.

Then, VPD, kPa:

VPD = SVP × (1 − RH/100)

Natural solar radiation was supplemented with artificial lighting to maintain a 15 h day and 9 h night schedule [11]. Plants were distributed in a completely randomized design along the greenhouse tables and were maintained in a well-watered condition during the initial pretreatment period. Seven replicate pots were established for each line, except for lines 19-31_15, which had very poor germination and was dropped from the experiment. The experiments were conducted in three sets from June to November 2022, as shown in Table 2.

### 4.2. Progressive Soil Drying

For all three sets of experiments, a soil dry-down experiment was initiated when plants had four fully developed trifoliate leaves. Before initiating the dry-down experiment, all the pots were irrigated until dripping in the afternoon and allowed to drain overnight. The next morning, pots were covered with two clear plastic bags, and a plastic tube was inserted between the base of the plant and the plastic bag to allow watering during the experiment [22]. The bag and the plastic tube were secured to the base of the stem with a plastic twist tie (Figure S1). Each pot was weighed immediately after covering, and the weight was recorded as the initial weight. Daily pot weights were measured between 1200 and 1400 Central Standard Time (CST) to determine gravimetric water loss due to transpiration. The daily transpiration rate (TR) was calculated as the difference in weight of each pot on successive days, as described by Devi et al. [8].

After the first three days of measuring daily TR, three pots of each line were assigned as well-watered (WW), and four pots were assigned as water-deficit stressed (DD). The WW plants were used as a reference to calculate the normalized transpiration rate (NTR). The well-watered plants were maintained at 100 g below the initial saturated pot weight by replacing the daily amount of water lost. The remaining four pots were subjected to gradual soil drying at a rate of no more than 70 g per day. The DD plants were watered only if the daily loss was greater than 70 g day^−1,^ as described by Shekoofa et al. [10]. After the experiment, the total transpirable soil water available to the plant of each pot was calculated as the difference between the initial and final weight of the pot. To compare lines, the availability of soil water was expressed as the fraction of transpirable soil water (FTSW) for each pot in the DD treatment every day. FTSW was calculated as the difference between daily and final weight, divided by the total transpirable water, as shown in the equation below:

FTSW = (daily weight − final weight)/(initial weight − final weight)

The use of transpirable soil water as the basis of comparing plant response to soil drying under a range of conditions has been effectively used in a number of studies [7,8]. The transpiration data were analyzed using the procedure described by Ray and Sinclair [7]. To minimize the influence of different plant sizes that cause significant variations in daily TR across days, the daily ratio was calculated between the TR of each drought-stressed pot divided by the average TR on that day for the well-watered plants within each line. Subsequently, a second normalization was made to facilitate comparison among soybean lines. This normalization was achieved by dividing the daily transpiration rate using the mean transpiration rate of the same plant during the first three days of the experiment when the soil was still at high water content. This ratio was identified as the normalized transpiration rate (NTR), and its value at the initial phase of the dry-down cycle for every plant was 1.0. The experiment was terminated when all the DD plants reached an NTR value below 0.10, which was defined as the endpoint of the transpirable soil water.

### 4.3. Statistical Analysis

The statistical analysis was conducted by calculating a two-segment linear regression analysis to plot the relationship between NTR and FTSW. The resulting graphs were generated using GraphPad Prism 8.0 (GraphPad Software Inc., San Diego, CA, USA). This software allows us to determine the threshold FTSW between the two segments where the NTR decrease was initiated. Treatment means were separated using Tukey’s honest significant difference (HSD) test (*p* < 0.05) (JMP, version Pro 16, SAS Institute Inc., Cary, NC, USA).

## 5. Conclusions

From this set of experiments, we can conclude that almost 65% of the RILs had FTSW threshold values between 0.41 and 0.80. These results support the earlier conclusion of Shekoofa et al. (2017) [17] that the frequency of expression of limited transpiration in progeny lines was reasonably high. Thus, crossing in a breeding program, with parents expressing drought tolerant traits (i.e., early stomatal closure and limited transpiration) may result in inheritance with a reasonably high percentage of RILs expressing a high FTSW threshold that is in a beneficial range to achieve drought tolerance in the field.

## Figures and Tables

**Table 1:** The number of data points (n), fraction of transpirable soil water (FTSW) threshold for initiation of decline in normalized transpiration rate (NTR) as determined using the two-segment, linear-regression analysis, confidence intervals (CIs) and R^2^ from the regression analysis for soybean lines in soil drying down experiments. Those thresholds identified with different letters were significantly different between lines.

	Lines	n	FTSW_threshold_	Confidence Interval (*p* < 0.05)	R^2^
**Set 1**	19-31_1	55	0.66	0.572–0.743 a	0.88
	19-31_2	53	0.55	0.498–0.595 ab	0.96
	19-31_3	53	0.63	0.568–0.700 a	0.92
	19-31_4	55	0.66	0.593–0.727 a	0.92
	19-31_5	53	0.48	0.423–0.535 b	0.91
	19-31_6	52	0.58	0.497–0.655 ab	0.88
	19-31_7	53	0.68	0.574–0.784 a	0.83
	19-31_8	52	0.56	0.509–0.609 ab	0.95
	19-31_9	40	0.65	0.583–0.717 a	0.94
	19-31_10	35	0.61	0.509–0.701 ab	0.87
**Set 2**	19-31_11	51	0.71	0.674–0.740 b	0.99
	19-31_12	70	0.64	0.595–0.690 b	0.94
	19-31_13	73	0.80	0.744–0.860 a	0.94
	19-31_14	59	0.61	0.570–0.649 b	0.96
	19-31_15 *	-	-	-	-
	19-31_16	46	0.72	0.661–0.779 ab	0.95
	19-31_17	60	0.66	0.613–0.710 b	0.95
	19-31_18	53	0.65	0.544–0.746 ab	0.85
	19-31_19	64	0.72	0.666–0.781 ab	0.93
	19-31_20	42	0.70	0.640–0.758 ab	0.97
**Set 3**	19-31_21	48	0.38	0.314–0.444 a	0.90
	19-31_22	48	0.21	0.186–0.239 b	0.93
	19-31_23	47	0.35	0.305–0.390 a	0.93
	19-31_24	49	0.18	0.156–0.199 b	0.94
	19-31_25	33	0.34	0.293–0.385 a	0.95
	19-31_26	46	0.25	0.211–0.297 ab	0.88
	19-31_27	46	0.40	0.344–0.462 a	0.89
	19-31_28	47	0.32	0.269–0.374 a	0.88
	19-31_29	48	0.22	0.185–0.253 b	0.90
	19-31_30	44	0.32	0.286–0.355 a	0.94

* Line 19-31_15 was excluded from the experiment due to poor seed germination.

**Table 2:** Summary of soybean lines, number of plant replications, and different environmental parameters in each set of experiments.

	Lines	Replications	Duration of Experiment	Day/Night Growth Temp (°C)	Temp Max (°C)	Temp Min (°C)
**Set 1**	19-31_1	7	13 d	40:30	43.0	22.8
	19-31_2	7	13 d	39:30	43.0	22.8
	19-31_3	7	13 d	38:30	43.0	22.8
	19-31_4	7	13 d	38:30	43.0	22.8
	19-31_5	7	12 d	39:30	43.0	22.8
	19-31_6	7	12 d	39:30	43.0	22.8
	19-31_7	7	13 d	40:30	43.0	22.8
	19-31_8	7	12 d	39:30	43.0	22.8
	19-31_9	7	13 d	39:30	43.0	22.8
	19-31_10	7	13 d	38:30	43.0	22.8
**Set 2**	19-31_11	7	13 d	28:22	37.0	11.5
	19-31_12	7	14 d	28:22	37.0	11.5
	19-31_13	7	13 d	28:22	37.0	11.5
	19-31_14	7	16 d	27:21	37.0	11.5
	19-31_15 *	-	-	-	37.0	-
	19-31_16	7	18 d	27:21	37.0	11.5
	19-31_17	7	15 d	27:22	37.0	11.5
	19-31_18	7	13 d	28:22	37.0	11.5
	19-31_19	7	16 d	27:21	37.0	11.5
	19-31_20	7	11 d	27:22	37.0	11.5
**Set 3**	19-31_21	7	11 d	30:23	44.5	17.5
	19-31_22	7	11 d	30:23	44.5	17.5
	19-31_23	7	11 d	30:23	44.5	17.5
	19-31_24	7	11 d	30:23	44.5	17.5
	19-31_25	7	11 d	30:23	44.5	17.5
	19-31_26	7	10 d	29:24	44.5	17.5
	19-31_27	7	11 d	30:23	44.5	17.5
	19-31_28	7	11 d	30:23	44.5	17.5
	19-31_29	7	11 d	30:23	44.5	17.5
	19-31_30	7	10 d	29:24	44.5	17.5

* Line 19-31_15 was excluded from the experiment due to poor seed germination.

## Data Availability

All data included in the main text and Supplementary Materials.

## Supplementary Materials

The following supporting information can be downloaded at: https://www.mdpi.com/article/10.3390/plants12183227/s1, Figure S1: Images show pots setup in the controlled environment (greenhouse). (Top) during the first four weeks of growth, (middle) during the initiation of dry-down, (bottom) when plants reached NTR = 0.10. Photo credit: The Shekoofa’ s lab.
