# Supplementary material for: Inheritance of Early Stomatal Closure Trait in Soybean: Ellis × N09-13890 Population
Source: Plants (Basel). 2023 Sep 11;12(18):3227. doi: 10.3390/plants12183227 (PMC10534556; doi:10.3390/plants12183227)
Supplement: Supplementary file 1 [file plants-12-03227-s001.zip › plants-2566743-supplementary.pdf]

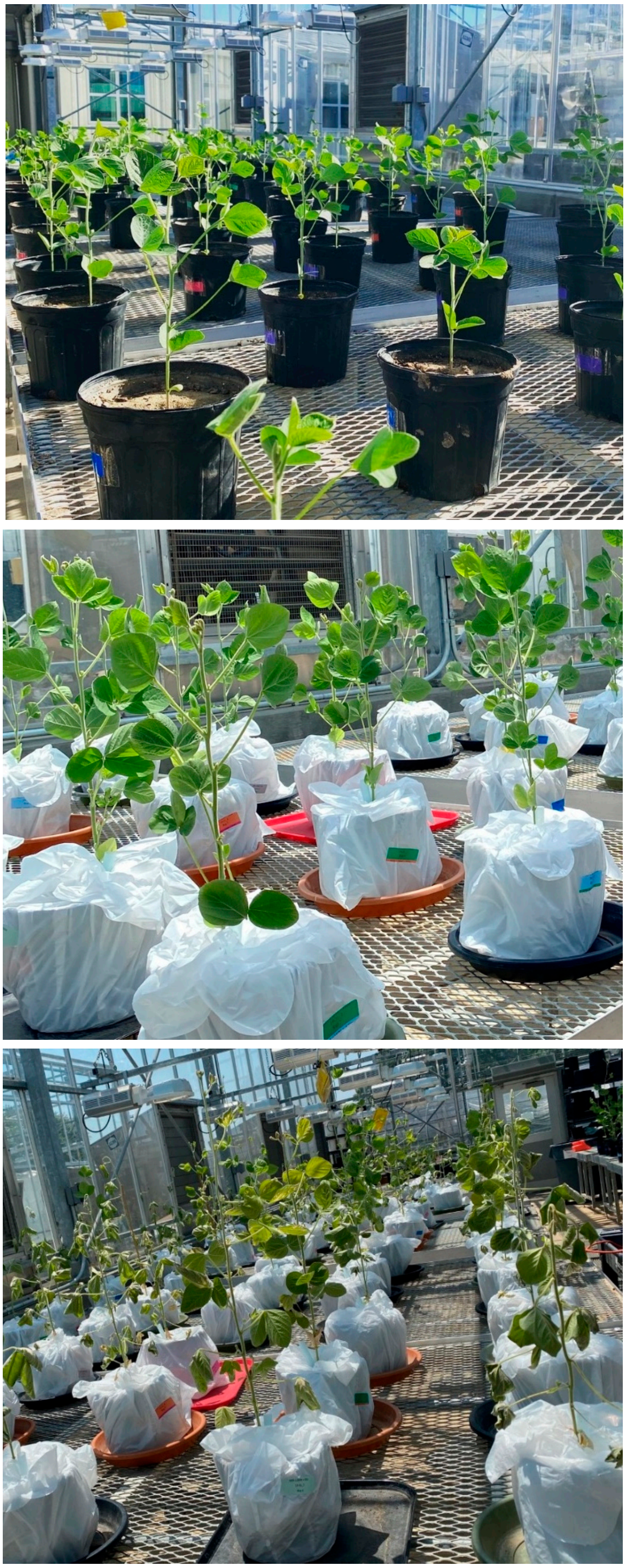

Figure S1.

Images show pots setup in the controlled environment (greenhouse). (Top) during the first four weeks of growth, (middle) during the initiation of dry-down, (bottom) when plants reached NTR= 0.10.

Photo credit: The Shekoofa' s lab
